# Time to adapt in the pandemic era: a prospective randomized non –inferiority study comparing time to intubate with and without the barrier box

**DOI:** 10.1186/s12871-020-01149-w

**Published:** 2020-09-14

**Authors:** Praneeth Madabhushi, Sudhakar Kinthala, Abistanand Ankam, Nitin Chopra, Burdett R. Porter

**Affiliations:** Department of Anesthesiology, Robert Packer Hospital, Guthrie Clinic, 1 Guthrie Square, Sayre, PA 18840 USA

**Keywords:** Barrier box, Airway management in COVID patients

## Abstract

**Background:**

The challenges posed by the spread of COVID-19 disease through aerosols have compelled anesthesiologists to modify their airway management practices. Devices such as barrier boxes are being considered as potential adjuncts to full PPE’s to limit the aerosol spread. Usage of the barrier box raises concerns of delay in time to intubate (TTI). We designed our study to determine if using a barrier box with glidescope delays TTI within acceptable parameters to make relevant clinical conclusions.

**Methods:**

Seventy-eight patients were enrolled in this prospective non-inferiority controlled trial and were randomly allocated to either group C (without the barrier box) or the study group BB (using barrier box). The primary measured endpoint is time to intubate (TTI), which is defined as time taken from loss of twitches confirmed with a peripheral nerve stimulator to confirmation of end-tidal CO _2._ 15 s was used as non-inferiority margin for the purpose of the study. We used an unpaired two-sample single-sided t-test to test our non- inferiority hypothesis (H _0_: Mean TTI diff ≥15 s, H _A_: Mean TTI diff < 15 s). Secondary endpoints include the number of attempts at intubation, lowest oxygen saturation during induction, and the need for bag-mask ventilation.

**Results:**

Mean TTI in group C was 42 s (CI 19.2 to 64.8) vs. 52.1 s (CI 26.1 to 78) in group BB. The difference in mean TTI was 10.1 s (CI -∞ to 14.9). We rejected the null hypothesis and concluded with 95% confidence that the difference of the mean TTI between the groups is less than < 15 s (95% CI -∞ to 14.9,*p* = 0.0461). Our induction times were comparable (67.7 vs. 65.9 s).100% of our patients were intubated on the first attempt in both groups. None of our patients needed rescue breaths.

**Conclusions:**

We conclude that in patients with normal airway exam, scheduled for elective surgeries, our barrier box did not cause any clinically significant delay in TTI when airway manipulation is performed by well-trained providers.

The study was retrospectively registered at clinicaltrials.gov (NCT04411056) on May 27, 2020.

## Background

The Coronavirus disease of 2019 (COVID-19) outbreak has presented unique challenges in airway management secondary to aerosol generation and transmission. Airway manipulation in a patient with COVID-19 is a high-risk procedure due to close proximity of the provider to the patients’ oropharynx [1]. Unrecognized asymptomatic and pre-symptomatic infections with SARS- CoV-2 virus might contribute to transmission [2]. Anesthesia Patient Safety Foundation (APSF) and American Society of Anesthesiologists (ASA) recommends as an optimal practice that all anesthesia professionals should utilize full personal protective equipment (PPE) appropriate for aerosol-generating procedures for all patients when working near the airway and use of video-laryngoscopy when possible in suspected patients [2]. Many predictive models suggest that the COVID-19 outbreak may last between 18 and 24 months [3]. Clinicians have been compelled to improvise and use protective barrier enclosures during airway manipulation to minimize aerosol exposure to operating room personnel [4]. Novel devices such as an “Intubation barrier box” are being mentioned as viable options when used during airway management. Based on online resources, we re-designed the barrier box to possibly provide additional protection to the personnel while safely providing airway management to our patients [5]. To our knowledge, this is the first clinical study to evaluate the impact of using the re-designed barrier box during airway management and its feasibility in routine clinical practice during this pandemic era.

The main objective of this study is to calculate the time to intubate (TTI) with our re-designed barrier box by well trained providers with prior simulation training in COVID negative patients needing elective surgery with normal airway assessment when used along with Glidescope® (Verathon, Bothell, WA, USA). TTI is defined as the time taken from loss of twitches confirmed with a peripheral nerve stimulator to the confirmation of end-tidal CO_2_ [6]. A non-inferiority design has been utilized to compare TTI with the barrier box in the study group vs control group (No barrier box) and conclude if the additional time taken for airway manipulation with a barrier box is within acceptable non-inferiority margin. Based on our clinical experience and pilot study data, we hypothesized that an additional 15 s of time (Δ = 15) with the box is considered acceptable to compare intubation techniques in both the groups [7]. We believe that the barrier box could be used safely during induction without clinically significant delay in TTI. (Mean difference of TTI < 15 s).

## Methods

This is a single-center, prospective non inferiority, controlled trial with balanced randomization [1:1], conducted at our institution. This study was approved by Institutional Review Board of The Guthrie Clinic, IRB00000918 **(**IRB# 2004–38) and retrospectively registered at clinicaltrials.gov (NCT04411056). Informed verbal consent was obtained, and the patients received a letter of invitation. Following institutional review board approval, patients presenting for surgery at our hospital were identified in the operating room schedule. Our study first patient recruitment was done on May 07,2020 and the last patient recruitment was done on May 19, 2020 .

In the pre-operative area, patients needing general anesthesia with endotracheal intubation were approached and enrolled in the study. All the patients over 18 years of age who were pre-operatively tested negative for SARS- CoV-2 virus were included in the study. Patient refusal, inability to consent or cooperate, children, pregnant women, patients with severe cardiopulmonary compromise, ASA physical status 4 and 5, Body Mass Index (BMI) > 35, known or anticipated difficult airway, patients with positive COVID status or unknown COVID status were excluded from the study. Patients who reported claustrophobia in the pre-operative area were also excluded from the study.

Sample size calculation was done by using the two means equation in the Power and Sample Size Calculation program (Dupont and Plummer, 1990) based on a study by Teoh et.al (The mean of intubation time for glidescope was 31.2 s and Airway Scope was 20.6 s). Based on a significant difference of either 10 s or 15 s between the groups,the two-tailed hypothesis testing concluded that each group needed 36 and 16 subjects respectively to achieve a power of 80%, with an error rate of 5% and standard deviation of 15 s [8, 7]. Upon further review, our research team decided to use 15 s as non-inferiority margin for our statistical analysis though the sample size of 36 patients in each group was used.

A pilot study with 20 patients helped us in calculating TTI (time to intubate) and enabled us to identify work-flow issues, the importance of checklist, and designating personnel with specific roles to troubleshoot problems effectively. (A checklist along with designated roles of airway team is available in supplementary reading material). Randomization was done by a research coordinator not directly involved in the study. The randomization schedule was placed in sealed envelopes and concealed from the researcher until the time of the informed verbal consent [9]. Patients were randomly assigned into control group C (without barrier box) and study group BB (with barrier box).

Only patients negative for the SARS-CoV-2 virus were included in this study to avoid unintentional exposure to operating room personnel and to conserve PPE. All providers participating in the study had at least three years of clinical experience in airway management and received training with the barrier box on a mannequin. The practical training with the mannequin was completed when the providers accomplished three successful intubations within 60 s for each attempt. A total of four anesthesiologists and four nurse anesthetists were involved in the airway management for the purpose of this study.

**Fig. 1:**
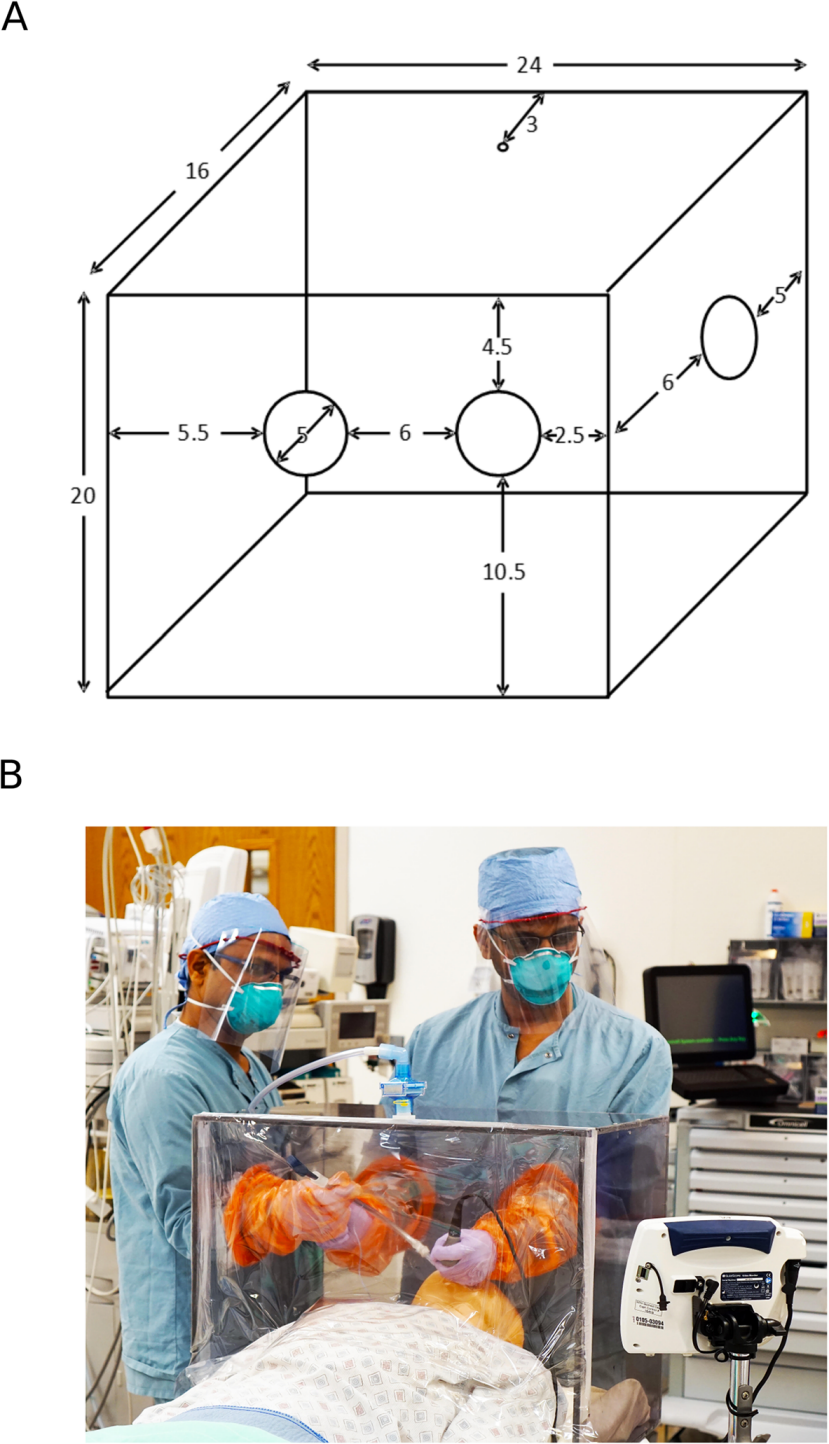
**a**: Schematic of the barrier box with dimensions (in inches). **b**: Airway team using Glidescope for intubation with the barrier boxes (Own Image)

An aerosol box was originally designed by Taiwanese doctor Hsien Yung Lai, with the intent to act as a barrier between aerosol and droplets generated at the patient’s airway and personnel performing aerosol-generating procedure [10]. Canelli et al. in their simulation found containment of aerosols and droplets within the box and suggested the box can be used as an adjunct to PPE’s [4]. Fig. 1 a shows the schematic of the barrier box with dimensions and Fig. 1 b shows the airway team intubating a mannequin with a barrier box (enclosed with a clear plastic drape on the front end) and glidescope. We made changes to this original design to make it more usable. Table 1 shows the modifications made to the original design and intended benefits.

**Table 1:** Difference between original box versus re-designed box and intended benefit

BOX Characteristics	Original BOX	Re-designed BOX	Intended benefit
Base	No	Yes	Improves stability
Width	20 in.	24 in.	To accommodate patient shoulders and position closer to the provider inside the box
Port diameter	4 in.	5 in.	To improve dexterity and range of motion
Port position	Equidistant from the sides, and at 10 in. from the base	Left port moved towards the center by 3 in. and the height of port increased to 13 in. from the base	Improves ergonomics for ease of intubation
Roof vent	None	Yes	To attach a filter and suction apparatus to minimize aerosol load
Long sleeve gloves	None	Yes	To seal the ports and minimize potential cross-contamination

Original Box –designed by Taiwanese doctor Hsien Yung Lai

After securing intravenous access in the pre-operative area, anxiolytics were given as per the discretion of the anesthesiologist caring for the patient. Upon arrival to the operating room, all patients were connected to the standard ASA monitors. A barrier box was placed at the head end of the operating table in the group BB, and the patient was positioned comfortably on a head-rest inside the barrier box. A transparent plastic screen with adhesive was attached to the open end of the box on three sides with the other remaining side lying on the patient. Patients in group C did not have a barrier box. A preprocedural checklist that included all the necessary airway equipment, medications, monitors, and suction was provided to the personnel involved in the study. We made sure all the equipment was functioning and ready to be used. A team of two providers were involved with anesthetic induction, one of them assigned to airway management and an assistant helping with medications, circuit connections, suctioning assistance with bag-mask ventilation, and stylet removal. The team used N95 masks and face shield while providing anesthetic care. The head end of the bed was elevated to 20 degrees measured with clinometer (phone-based app) [11]. Pre-oxygenation was achieved with supplemental oxygen via a mask for three minutes of tidal volume breaths [12]. A standard rapid sequence induction and intubation technique was performed in both the groups. All patients received intravenous induction doses of lidocaine 1–1.5 mg/kg, fentanyl 1–2 mcg/kg and propofol 1–2.5 mg/kg. After confirming the loss of consciousness, succinylcholine 1.5 mg /kg was given to achieve neuromuscular blockade [13]. Apneic oxygenation was continued after induction [14]. A peripheral nerve stimulator was used to confirm the loss of twitches. The designated provider managing the airway proceeded with intubation. A glidescope with styleted ETT was used for the intubation of all the patients. Endo-tracheal intubation was confirmed with positive end tidal CO_2_. If intubation was unsuccessful by the primary provider, further attempts by any other provider were documented separately. If difficulty to intubate is deemed secondary to the barrier box, it was immediately removed.

Our primary endpoint (TTI) was collected by an independent provider (registered nurse) not directly involved in airway management. The TTI was used as an outcome measure as it is an objective variable that can be measured and helps us in assessment of airway manipulation with a barrier box. We used objective end points to eliminate inter- observer variability. The premise of our original non-inferiority study is to compare the mean TTI and infer if the additional time taken for airway manipulation with a barrier box is within acceptable non-inferiority margin between the means of both the groups (Box vs No Box). Non- inferiority margin of 15 s was considered acceptable while comparing airway manipulation in both the groups based on our clinical experience and pilot study data. Our literature review showed that this non-inferiority margin (Δ = 15 s) would be within safe period of apnea tolerated by most patients [15]. Our secondary endpoints were total induction time, number of attempts at intubation, and lowest saturations observed during anesthetic induction and need for BMV. Induction time is defined as the time taken from propofol bolus to loss of twitches measured with a peripheral nerve stimulator.

This manuscript adheres to CONSORT updated guidelines for non-inferiority trial.

### Statistical analysis

R 3.5.3 software was used for statistical analysis. TTI was the primary endpoint in our study. Fifteen seconds was used as non-inferiority margin(Δ) for the purpose of this study. The mean time to intubate (TTI) of BB group was always expected to be higher than mean TTI of control group. Hence, a one-sided t- test was adapted to determine if there is a difference between groups in one specific direction. We hypothesized that the use of the barrier box prolongs the mean TTI by more than 15 s compared to group C (H_O_: Mean TTI diff ≥15 s, H_A_: Mean TTI diff < 15 s). We conducted two independent sample comparison of means using one sided t-test in R. The t-test was done based on assumptions that the sample has no dependence between subjects in groups C and BB (by study design), variance in both the groups are similar (confirmed by Levine’s test), and samples are drawn from a population with a normal distribution (sample size > 30). Results were considered statistically significant when *p* < 0.05.

## Results

**Fig. 2:**
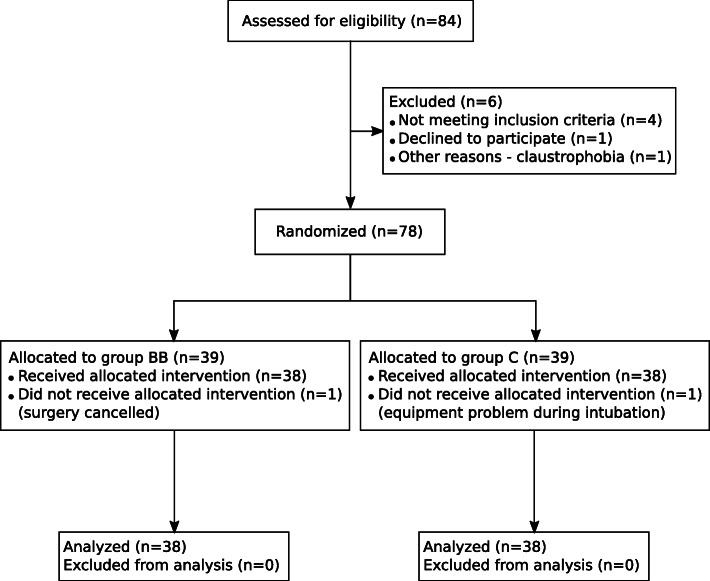
CONSORT Flow Diagram

A total of 84 patients were approached for the study. Figure 2 shows CONSORT flow diagram**.** One patient refused to be in the study, and the other opted out because of claustrophobia. Four patients did not meet inclusion criteria (two patients had a BMI > 35 recorded on the day of surgery, two SARS-CoV-2 had results pending). The remaining 78 patients were enrolled in the study and randomized. One case was canceled after arrival to the operating room prior to induction secondary to surgical equipment malfunction. One patient in the control group was excluded secondary due to equipment malfunction during intubation. Data for 76 patients were analyzed (38 in each group). Baseline demographics are comparable in both the groups and are presented in Table 2. Airway assessment was comparable in both the groups and is shown in Table 3.

**Table 2:** Demographic data and ASA Physical status in both the groups

Characteristics	Group C (***N*** = 38)	Group BB (N = 38)
**Age** mean (SD) in years	52.7 (18.9)	57.6 (16.7)
**Sex** (male/female)	18/20	19/19
**BMI** mean (SD)	29 (4.3)	28.7 (4.1)
**ASA 1 (N)**	2	2
**ASA 2 (N)**	25	21
**ASA 3 (N)**	11	16

*Abbreviations: ASA* American Society of Anesthesiology*, BMI* Body mass index*, SD* Standard deviation*, N* number of patients

**Table 3:** Airway assessment data in both the groups

Characteristics	Group C (N = 38)	Group BB (N = 38)
Mallampati (N)	3	3
Mallampati 2 (N)	31	29
Mallampati 3 (N)	4	6
TMD > 3 Finger breadth	38	38
Inter incisor distance > 3 finger breadth	38	38
Neck range of motion (Full/Restricted)	38/0	36/2

*Abbreviations: TMD* thyromental distance*, N* number of patients

Data comparing primary and secondary end points is shown in Table 4. Mean TTI in group C was 42 (CI 19.2 to 64.8) seconds vs 52.1 (CI 26.1 to 78) seconds in group BB. The difference in mean TTI was 10.1 s (CI -∞ to 14.9). We rejected the null hypothesis and concluded with 95% confidence that the difference of the mean TTI between the groups is less than < 15 s (d = 0.7,95% CI -∞ to 14.9,*p* = 0.0461). Based on our results, the delay in TTI with barrier box (mean diff < 15 s) falls within the safe period of apnea tolerated by most patients. Our induction times were comparable (67.7 s (CI 43.7 to 91.7) in group C vs 65.9 s (CI 39.2 to 92.6) in group BB). 100% of our patients were intubated on the first attempt in both the groups. None of our patients needed rescue breaths. SpO_2_ < 90% was observed in two patients, one in each group with immediate improvement in saturation after the placement of ETT. Table 3 shows our primary and secondary end points. We observed one patient with significant bradycardia following induction in the control group that improved spontaneously prior to intubation.

**Table 4:** Primary and secondary end points of the study

End Point	Group C (N = 38)	Group BB (***N*** = 38)
TTI Mean (95% CI) in seconds	42 (19.2 to 64.8)	52.1 (26.1 to 78)
Induction Time Mean (95% CI) in seconds	67.7 (43.7 to 91.7)	65.9 (39.2 to 92.6)
Lowest saturation during induction SpO_2_ (Mean)	98.7	98.8
Need for BMV (During induction)	0	0
Intubation in first attempt (%)	100	100

*Abbreviations: TTI* Time to intubate*, BMV* Bag Mask ventilation*, SD* Standard deviation*, SpO*_*2*_ peripheral capillary oxygen saturation*, N* number of patients

## Discussion

To our knowledge, this is the first randomized clinical trial evaluating the use of a barrier box. Providing safe anesthetic care to our patients as well as protecting operating room personnel is of paramount importance during this pandemic. The goal of an optimal airway management is to minimize the interval between induction and successful ETT placement without compromising patient safety. We tested the impact of using the barrier box during airway manipulation when used in conjunction with video-laryngoscopy.

We performed adequate pre-oxygenation and apneic oxygenation to extend the safe apnea period, which is defined as the time between the onset of apnea and when the SpO_2_ reaches 90% or less [14]. This provides valuable additional time to secure the airway. Our results found that the barrier box did not prolong TTI in non–morbidly obese (BMI < 35) patients with normal airway assessment to cause any clinically significant hypoxia and falls within the safe period of apnea tolerated by most patients [15–17]. Our secondary endpoints show that the box did not increase the number of attempts at intubation. None of the patients needed bag-mask ventilation prior to intubation, which is essential during this pandemic era as bag-mask ventilation can increase the aerosol generation.

A recent study by Begley et al. described the significant challenges while intubating a mannequin when a barrier box was used [18]. After using the original box on a mannequin and patients in the pilot study, we made modifications to the original design to improve dexterity and usability of the box. Most providers were comfortable after 3–5 intubations with the box. The providers involved in the study were well trained and experienced to perform under stressful situations. We believe that two dedicated anesthesia personnel with designated roles are needed to perform airway management safely while using the barrier box. If rescue ventilation is needed, it usually involves a two-person technique. A plan was in place for removal of the box if needed. Removal of the box does require two people, with the assistant standing on the patient’s side, holding the patient head with the headrest, while the operator slides the box away from the operating table. An unanticipated difficult airway could create anxiety, in which case our box could be safely removed.

The barrier box was made of polycarbonate, which was heavy, but provided stability and could be moved around by a single person. However, if the usage is widely adapted, manufacturers could assemble the barrier boxes with lighter material. A vent located on the roof was designed to connect to suction via a high-efficiency filter to possibly reduce the aerosol load. This feature was not utilized for our study. Long sleeve gloves were used to seal the ports and serve as barriers to prevent cross-contamination. Though our study is not designed to evaluate the integrity of PPE while using the barrier box, we have not observed any breach to long sleeves gloves. Regular gloves were used over the long sleeve gloves to provide a better tactile feel. We also developed a process of discarding the disposable gloves and cleaning the barrier box after use. Barrier boxes were only transported after completion of the cleaning process in the operating room. Our study was limited to anesthetic induction and intubation, but the usage of the barrier box can be extended to emergence where the potential for aerosol generation is higher. We did not study its impact during the time of emergence.

Our study has a few limitations. The results of our study may not apply to other barrier box designs. Since the time we started using our re-designed barrier box we have encountered multiple designs on internet websites. There is scope for improvement in design, efficacy and contamination safety of the box which needs to be addressed in future studies. The providers who participated in the study had at least 3 years of clinical experience in airway management and all of them received simulated training with the barrier box prior to intubating a patient. We did not test this barrier box when used by inexperienced or non–anesthesia providers. However, it is recommended that the most experienced provider perform intubation in COVID patients to minimize the number of attempts [2].

By using a suction connected via a HEPA filter, the aerosol load inside the box could potentially be reduced. Though this concept is theoretical, our research in no way could establish the concept, nor prove its utility. Once the efficacy of the box is established beyond doubt, additional features like suction connections might become a useful tool. We did not delve into this as it is beyond the scope of our research. Further studies need to be conducted to evaluate the effectiveness and the integrity of the PPE when used with the barrier box. Though we did not study the box during emergence, the barrier box can pose potential safety issues with a restless patient especially if they try to sit up. Also, we noticed that the height of the box could interfere when holding the styleted ETT, in a vertical position in some patients. We believe increasing the height by 6 in. could help to accommodate the styleted endotracheal tube while intubating as well as a restless patient trying to sit up. Barrier box may be difficult to use in an uncooperative patient and should be avoided.

We understand that the effectiveness of the barrier box has been a much-debated topic in recent times. However, our trial was not designed to study the effectiveness of the barrier box in minimizing aerosol exposure. The barrier box was not tested in patients with BMI > 35, where airway management could be challenging. A barrier box could be an additional source of anxiety when providing care for high-risk patients, where delay in intubation can lead to clinically significant desaturation. Based on our findings, we cannot make any recommendations regarding the use of the barrier box in patients with limited cardiopulmonary reserve and warrants further research. If a flexible fiberoptic bronchoscope is needed,we recommend removing the box.

## Conclusions

We conclude that in COVID negative patients who are not morbidly obese (BMI < 35) with normal airway exam, scheduled for elective surgeries, our re-designed barrier box did not cause any clinically significant delay in TTI when airway manipulation is performed by well-trained providers. We believe an ergonomically designed barrier box complemented with a pre-procedure checklist and a well-prepared team can enhance the safety and the success of intubation. The use of a barrier box could be extended to any patient with a potential aerosol transmissible infection. Any proposed design or equipment requires testing and re-testing for validation, and we believe our study provides early steps in this direction.

## Supplementary information

**Additional file 1.**

**Additional file 2.**

## Data Availability

Raw data submitted as an excel sheet in supplementary reading material.

## Abbreviations

COVID-19: Coronavirus disease of 2019
ASA: American Society of Anesthesiologists
APSF: American Patient Safety Foundation
PPE: Personal protective equipment
TTI: Time to intubate
GROUP BB: Group Barrier Box
GROUP C: Group Control
ETT: Endotracheal tube
BMI: Body Mass Index
BMV: Bag-Mask Ventilation
TMD: Thyromental distance
