# Supplementary material for: Time to adapt in the pandemic era: a prospective randomized non –inferiority study comparing time to intubate with and without the barrier box
Source: BMC Anesthesiol. 2020 Sep 14;20:232. doi: 10.1186/s12871-020-01149-w (PMC7488639; doi:10.1186/s12871-020-01149-w)
Supplement: Supplementary file 1 — Additional file 1. [file 12871_2020_1149_MOESM1_ESM.pdf]

CHECK LIST FOR INTUBATION USING A BARRIER BOX

- ☐ IV site - in working condition
- ☐ ASA monitors on
- ☐ Peripheral nerve monitor –working condition
- ☐ Head-end elevated by 20 degrees
- ☐ Medications with correct dosing and flush available
- ☐ Glidescope in working condition
- ☐ Airway adjuvants – oral, nasal airway and LMA readily accessible
- ☐ Styleted Endotracheal tube with syringe – cuff checked
- ☐ Eye tape and ETT tape
- ☐ Long sleeve gloves pre-taped and nitrile gloves ready
- ☐ Functioning suction
- ☐ Pre-oxygenation -Oxygen flowing

Role of Intubating Specialist

- Pre oxygenation
- Ventilation with two-person technique- Hold the mask (if rescue breaths are needed)
- Intubation using glidescope
- Securing ETT
- Eye protection tape
- Remove the box in case of emergency

Role of Assisting Specialist (Stand on right side of the patient)

- Prepare Box with pre –taped long sleeve gloves
- Administration of medications
- Ventilation with two-person technique- Hold the Bag (for rescue breaths)
- Removing the glidescope stylet following intubation
- Stabilize patient’s head while the box is removed in an emergency
- Remove the box following intubation
- Proper disposal of the contaminated gloves, plastic screen, disinfection and transport the box
