# Supplementary figures and images for: Time to adapt in the pandemic era: a prospective randomized non –inferiority study comparing time to intubate with and without the barrier box
Source: BMC Anesthesiol. 2020 Sep 14;20:232. doi: 10.1186/s12871-020-01149-w (PMC7488639; doi:10.1186/s12871-020-01149-w)

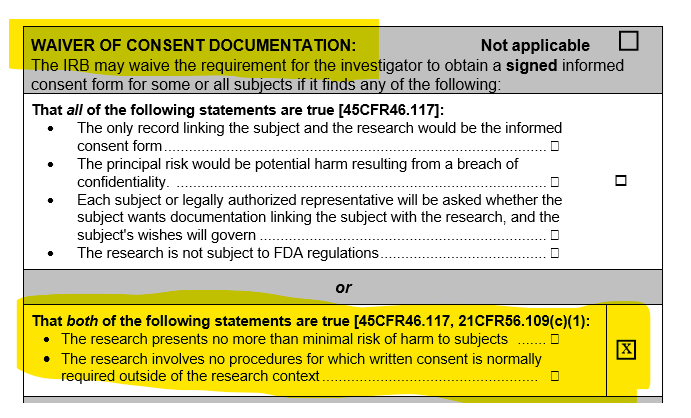

Supplement: Supplementary file 2 — Additional file 2. [file 12871_2020_1149_MOESM2_ESM.png]
